# A dysflagellar mutant of *Leishmania (Viannia) braziliensis *isolated from a cutaneous leishmaniasis patient

**DOI:** 10.1186/1756-3305-5-11

**Published:** 2012-01-11

**Authors:** Rogéria C Zauli, Jenicer KU Yokoyama-Yasunaka, Danilo C Miguel, Alexandre S Moura, Ledice IA Pereira, Ildefonso A da Silva, Lucianna GN Lemes, Miriam L Dorta, Milton AP de Oliveira, André N Pitaluga, Edna AY Ishikawa, Juliany CF Rodrigues, Yara M Traub-Cseko, A Tania Bijovsky, Fátima Ribeiro-Dias, Silvia RB Uliana

**Affiliations:** 1 Departamento de Parasitologia, Instituto de Ciências Biomédicas, Universidade de São Paulo, São Paulo, 05508-900, Brasil; 2 Instituto de Patologia Tropical e Saúde Pública, Universidade Federal de Goiás, Goiânia, 74605-050, Brazil; 3 Laboratório de Biologia Molecular de Parasitas e Vetores, Instituto Oswaldo Cruz, Fiocruz, Rio de Janeiro, 21045-900, Brazil; 4 Núcleo de Medicina Tropical, Universidade Federal do Pará, Belém, 66055-240, Brazil; 5 Laboratório de Ultraestrutura Celular Hertha Meyer, Instituto de Biofísica Carlos Chagas Filho, Universidade Federal do Rio de Janeiro, Rio de Janeiro, 21944-970, Brazil; 6 Universidade Federal do Rio de Janeiro, Pólo Avançado de Xerém, Rio de Janeiro, Brazil

**Keywords:** flagellum, mutant, *Leishmania*, electron microscopy.

## Abstract

**Background:**

Parasites of the *Leishmania *genus alternate between the flagellated extracellular promastigote stage and intracellular amastigotes. Here we report the characterization of a *Leishmania *isolate, obtained from a cutaneous leishmaniasis patient, which presents peculiar morphological features.

**Methods:**

The parasite was cultured *in vitro *and characterized morphologically using optical and electron microscopy. Identification was performed based on monoclonal antibodies and internal ribosomal spacer typing. *In vitro *macrophage cultures, murine experimental models and sand fly infections were used to evaluate infectivity *in vitro *and *in vivo*.

**Results:**

The isolate was identified as *Leishmania *(*Viannia*) *braziliensis*. In the atypical promastigotes grown in culture, a short flagellum surrounded or interrupted by a protuberance of disorganized material was observed. A normal axoneme was present close to the basal body but without elongation much further outside the flagellar pocket. A disorganized swelling at the precocious end of the axoneme coincided with the lack of a paraflagellar rod structure. The isolate was able to infect macrophages *in vitro*, induce lesions in BALB/c mice and infect *Lutzomyia longipalpis*.

**Conclusions:**

Notwithstanding the lack of an extracellular flagellum, this isolate infects macrophages *in vitro *and produces lesions when inoculated into mice. Moreover, it is able to colonize phlebotomine sand flies. Considering the importance attributed to the flagellum in the successful infection and survival of *Leishmania *in the insect midgut and in the invasion of macrophages, these findings may bring new light into the infectious mechanisms of *L*. (*V*.) *braziliensis*.

## Background

*Leishmania *spp. are the etiological agents of a group of diseases known as leishmaniasis. The complex clinical presentations vary from localized cutaneous lesions to fatal visceral involvement. Leishmaniasis is endemic in the whole tropical and subtropical world with estimates of 12 million people currently infected. This protozoan parasite undergoes a complex life cycle alternating between the phlebotomine sand fly vector and a mammalian host. The vector is infected by the ingestion of a contaminated bloodmeal containing amastigotes. In the insect midgut, parasites transform into promastigotes, spindle-shaped, highly motile forms with a single flagellum that emerges from the anterior flagellar pocket. Inside the insect's gut, *Leishmania *parasites differentiate into several distinct developmental stages until they finally transform into metacyclic promastigotes. Each of these stages is characterized by morphological and functional changes that warrant their survival in the fly. After blood digestion and degradation of the peritrophic membrane the parasites attach to the gut epithelium where they multiply. In the case of Old World sand flies and parasites the attachment is mainly due to interactions provided by the major promastigote surface molecule, the lipophosphoglycan (reviewed in [1]). The mechanism is not so clear in New World vector-parasite pairs, but might involve lectins (reviewed in [2]). The differentiation into infective metacyclic promastigotes coincides with the detachment from the gut and migration towards the stomodeal valve [1]. Flagellar length varies from 10 to 20 micrometers in promastigotes [3]. In addition to conferring motility to the promastigote, the flagellum has been shown to be involved in the attachment to the gut in the female phlebotomine sand fly and to environmental sensing [3-6]. In some instances, the lack of flagellum was associated with failure to survive in the phlebotomine fly [7].

Transmission to the vertebrate host, including wild reservoirs, domestic animals and humans, occurs during the insect bite by the inoculation of metacyclic promastigotes into the skin. These are engulfed by mononuclear phagocytic cells and transform into amastigotes, round-shaped forms lacking an external flagellum. The invasion of macrophages by *Leishmania (L.) donovani *has also been related to flagellar attachment [8].

*L. (V.) braziliensis *is the most common *Leishmania *species in the Americas and the most important causative agent of cutaneous and mucocutaneous leishmaniasis in Brazil [9]. In this paper, we describe a morphologically atypical *Leishmania *isolate, obtained from a cutaneous leishmaniasis patient in Brazil.

## Methods

### Parasites

The MHOM/BR/2006/EFSF isolate, referred herein as EFSF6, was obtained from a patient attending the Anuar Auad Tropical Diseases Hospital in the city of Goiânia, Goiás, Brazil. The patient was infected in Minaçu, a municipality of Goiás State and presented five ulcerated lesions distributed in both arms. The lesions had appeared three months before the diagnosis. The patient was cured after treatment with a 20-day course of pentavalent antimonial. As part of the diagnostic procedure, skin biopsies were taken and the tissue was homogenised and inoculated into Grace's medium (Sigma- Aldrich Chem. Co., St Louis, MO, USA) supplemented with 20% fetal calf serum (FCS) [10].

The isolate was typed by PCR of ribosomal DNA, glucose-6-phosphate dehydrogenase and *META2 *gene as described [11-13] and identified as *L. (V.) braziliensis*. Soon after isolation, the culture was frozen and further tests were performed with freshly recovered cultures with a low passage number (third to seventh).

Atypical promastigotes of the EFSF6 isolate or typical forms of the *L. (V.) braziliensis *MHOM/BR/75/M2903 reference strain were grown in M199 liquid medium (Sigma-Aldrich) supplemented with 10% fetal calf serum (FCS; Invitrogen) and 2% sterile male human urine, or in Grace's medium supplemented with 20% FCS and 2 mM L-glutamine (Sigma) and incubated at 22-26°C.

Analysis of the parasite's morphology by optical microscopy was performed by applying and spreading slightly 10 μl of culture onto a slide. The material was left to dry at room temperature and fixed and stained with the Instant Prov kit (Newprov, Pinhais-Paraná, Brazil).

### Monoclonal antibody typing

For slide preparation, promastigotes were centrifuged at 1000 × *g *for 10 min, washed once in PBS pH 7.2 (2.5 mM NaH_2_PO_4_, 7.4 mM Na_2_HPO_4_, 137 mM NaCl), applied to the slides, dried and fixed with acetone for 15 min.

The following monoclonal antibodies were used: B2, B5, B11, B12, B18, B19, M2, T3, CO2, L1, WIC, W1, W2, N2, N3 and WA2, according to methods described previously [14-16]. The B and N series react with species of the subgenus *L. (Viannia)*; M2, T3, WIC.79.3, W1, W2 and WA2 react with parasites of the subgenus *L. (Leishmania)*. CO2, and L1 are group-specific and react with members of subgenera *Viannia *and *Leishmania, Endotrypanum *and some species of the genus *Trypanosoma*. The monoclonal B18 is specific for *L. (V.) braziliensis*.

### Ribosomal Internal Transcribed Spacer (ITS) amplification and cloning

Genomic DNA was obtained from cultured parasites as described previously [17]. Amplification of the ribosomal DNA internal transcribed spacers 1 and 2 (ITS1, ITS2) and 5.8S ribosomal DNA (5.8S) (approximately 1 kb) was performed with primers IR1 (5' GTA GGT GAA CCT GCA GCA GCT GGA TCA TT 3') and IR2 (5' GCG GGT AGT CCT GCC AAA CAC TCA GGT CTG 3') or 5.8R (5'GGA AGC CAA GTC ATC CAT C 3') as described [18]. Amplified products were cloned into TOPO TA^® ^(Invitrogen). GenBank accession numbers for sequences determined or used in this study are listed in the legend of Additional File 1.

Nucleotide sequences were analyzed using the program BioEdit http://www.mbio.ncsu.edu/BioEdit/bioedit.html and aligned with sequences from GeneBank using ClustalW [19]. The resulting alignments were manually refined. Parsimony and bootstrap analyses were carried out using PAUP* 4.0 [20] with 100 replicates of a random addition sequence followed by branch swapping (RAS-TBR), as previously described [21].

### Infection of macrophages *in vitro*

Bone marrow-derived macrophages (BMDM) were obtained from BALB/c mice as described by [22]. BMDM were counted and distributed in 24-well plates on round coverslips (3 × 10^5 ^macrophages per well) containing RPMI 1640 medium with 10% FCS and 5% L929 cell conditioned medium and allowed to adhere overnight at 37°C, at 5% CO_2_. Infections of macrophages were performed using ratios of 15 stationary-phase promastigotes per macrophage. Infected macrophage cultures were kept at 33°C, 5% CO_2 _for 3 h in RPMI 1640 medium with 10% FCS and then washed twice with sterile PBS to remove free promastigotes. After 48 h of incubation, slides were fixed in methanol and stained with the Instant Prov kit (Newprov, Pinhais-Paraná, Brazil) for subsequent examination under light microscopy.

### Infection of mice

Female BALB/c mice (n = 5 - 7) were infected in the left ear by intradermal inoculation of 1 × 10^5 ^stationary-phase promastigotes grown in M199 liquid medium. Stationary-phase promastigotes (5 × 10^6^/50 μL of PBS) grown in Grace's medium were injected into the hind left footpads of C57BL/6 and BALB/c mice (n = 6). During 12 weeks, the evolution of lesions was evaluated by measuring the thickness of the infected ear or footpad using a calliper.

### Electron microscopy

Logarithmic-phase promastigotes (5 × 10^7^/mL), washed twice with PBS pH 7.2, or tissue fragments, were fixed in a solution containing 2.5% glutaraldehyde in 0.1 M cacodylate buffer, pH 7.2, for 24 h at 4°C. After fixation, the material was washed twice in 0.1 M sodium cacodylate buffer, pH 7.2 and post-fixed for 30 min in a solution containing 1% OsO_4_, 1.25% potassium ferrocyanide and 5 mM CaCl_2 _in 0.1 M cacodylate buffer, washed in the same buffer, dehydrated in acetone series, and embedded in Epon or Spurr resin. Ultrathin sections were obtained in a Leica Ultramicrotome, stained with uranyl acetate and lead citrate and observed under a Zeiss 900 or a JEOL 100 CX transmission electron microscope operating at 80 kV. Images were recorded with a MegaView III camera (Olympus Soft Imaging Solutions) using the ITem Software. For scanning electron microscopy, cells were fixed as before, adhered to poly-L-lysine-coated coverslips, post-fixed for 30 min in a solution containing 1% OsO_4_, 1.25% potassium ferrocyanide and 5 mM CaCl_2 _in 0.1 M cacodylate buffer, washed in the same buffer, dehydrated in ethanol series, critical point dried, and coated with gold in a Balzers gold sputtering system. Cells were observed under a Jeol JSM 6340F field emission scanning electron microscope operating at 5 kV.

### Rearing of Sand flies and artificial infection

*L. longipalpis *were captured at Gruta da Lapinha, Minas Gerais, Brazil, and maintained in an insectary at Instituto Oswaldo Cruz, FIOCRUZ. Capture, maintenance and colonization in laboratory conditions were performed according to Brazil and Brazil [23]. Insects were sugar fed on 30% sucrose solution *ad libitum*.

For artificial blood-feeding or infection with the EFSF6 isolate or typical forms of the *L. (V.) braziliensis *MHOM/BR/75/M2903, three-day-old female sand flies were fed through chick skin membrane on hamster blood containing 10^7 ^promastigotes/ml.

### Quantification of infection

DNA was extracted from individual insects collected at 24, 72 or 96 hours post artificial infection with *L. (V.) braziliensis *MHOM/BR/75/M2903 or EFSF6 using wizard SV Genomic kit (Promega). Parasite load was assessed as described before [24]. qPCR reactions were performed using the SYBR Green PCR Master Mix (Applied Biosystems) and kDNA [25] and 18S primers [24]. Standard curves were constructed using serial dilutions of *L. braziliensis *and *L. longipalpis *DNA. The relative quantification was calculated using the 2^-ΔΔCt ^method [26]. The Ct values of the 18S reference gene from *L. longipalpis *were used for data normalization.

### Ethical aspects

This study was approved by the Ethics Committee in Human and Animal Research of the HC/UFG and a consent letter was signed by the patient. All animal procedures were approved by the FIOCRUZ bioethics committee (CEUA - protocol number P0-116-02) and by ICB-USP Ethics Committee on Animal Experimentation.

### Statistical analysis

Results are presented as media ± SEM and data were analyzed by GraphPad Prism 4.0 Software (San Diego, CA, USA), using the Kruskal-Wallis and Mann-Whitney tests (p < 0.05).

## Results

### Isolation of EFSF6 parasites and preliminary morphological characterization

**Figure 1:**
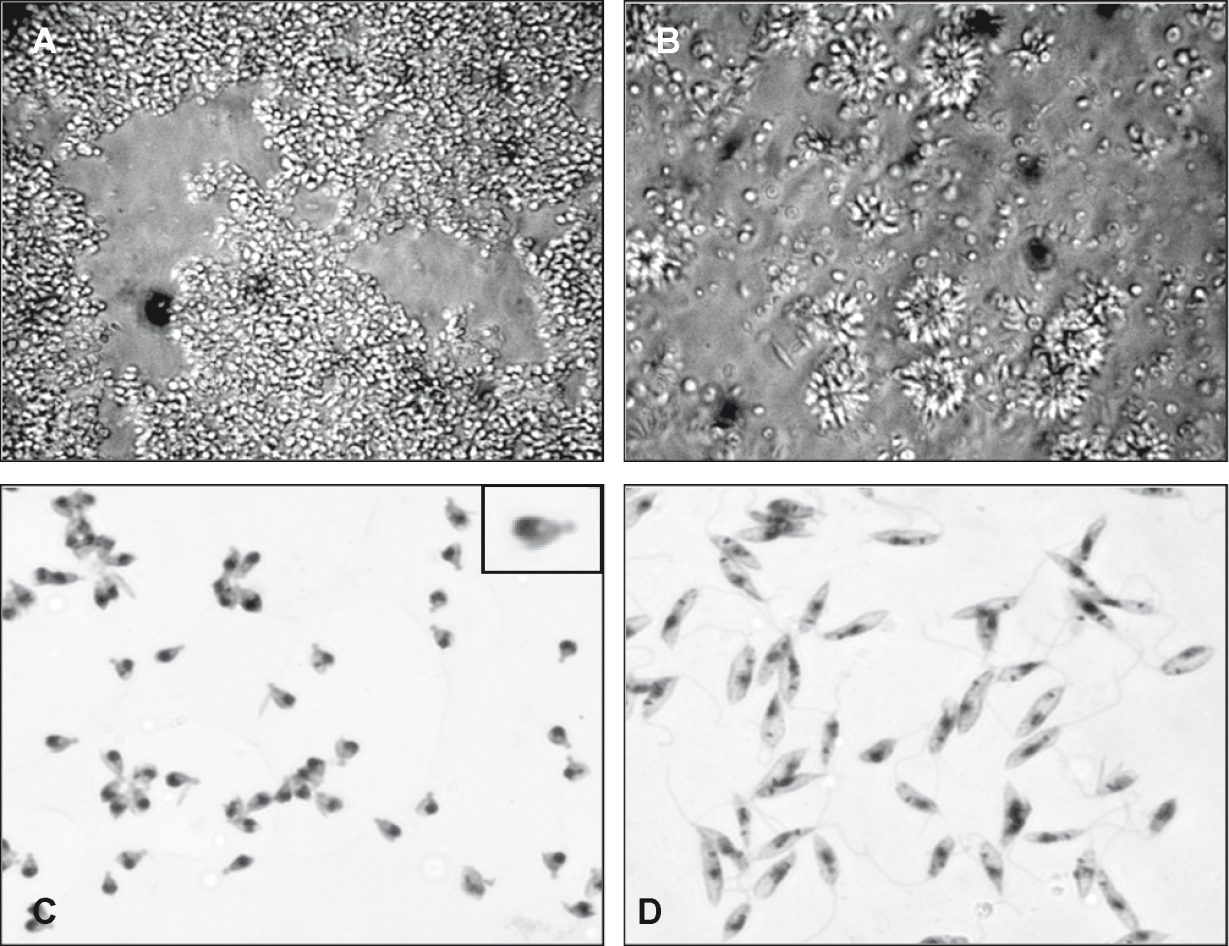
**Morphology of the EFSF6 isolate**. Phase contrast image (A, B) of EFSF6 (A) and M2903 (B) live promastigote cultures; magnification 400×. Microphotographs of stained EFSF6 (C) and M2903 (D) promastigotes; magnification 1,000×. Box in (C) shows the atypical flagellum (arrow).

EFSF6 parasites were initially obtained from skin biopsy homogenates cultured in Grace's medium. The observation of EFSF6 parasites recovered in primary *in vitro *cultures revealed an unusual morphology, distinct from conventional promastigotes: under phase-contrast, parasites looked round and were deposited at the bottom of the flasks (Figure 1A), while usual promastigotes appear as motile forms sometimes grouped in rosettes, as exemplified by the *L. (V.) braziliensis *reference strain M2903 (Figure 1B). Motility of EFSF6 parasites was greatly diminished as compared to M2903 cultures. While flagellated promastigotes exhibited a continuous oscillation pattern accompanied by rapid dislocation across the field of some parasites, EFSF6 cells only exhibited a fine vibrating pattern, without any significant displacements (Additional Files 2 and 3).

Optical microscopy revealed that most EFSF6 cells were round and presented a small and round protrusion at the body surface in the anterior region (Figure 1C), which was reminiscent of the rudimentary flagellum observed in the amastigotes. About 2-3% of EFSF6 parasites observed under optical microscopy presented a visible short flagellum, extending from the anterior end of the cell while all M2903 promastigotes were flagellated cells (Figure 1D).

**Figure 2:**
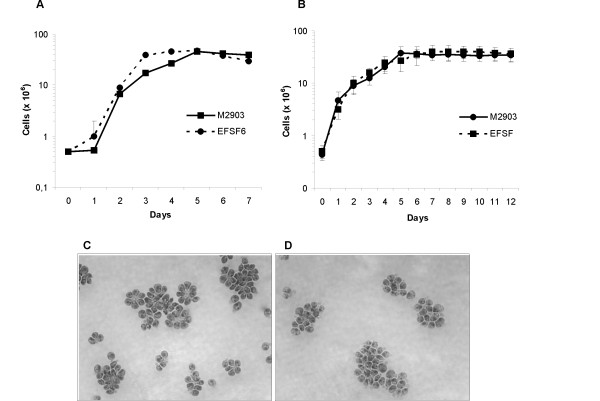
**Growth curves of EFSF6 and M2903**. Promastigotes cultivated in medium 199 with 10% FCS and 2% sterile human male urine (A) or in Grace's media with 20% FCS (B). Logarithmic-phase (C) and stationary-phase (D) EFSF6 promastigotes in Grace's media; magnification 1000×.

The growth pattern of EFSF6 promastigotes was analysed using two different culture media. Both in Grace's and M199 the EFSF6 parasites were able to multiply and reach stationary-phase at a similar rate and to the same density as the control *L. (V.) braziliensis *M2903 strain (Figure 2A and 2B). The morphology of EFSF6 stationary-phase parasites remained unaltered when compared to that observed for logarithmic-phase promastigotes (Figure 2C, D).

We also tested whether promastigote morphology varied with temperature. Varying the incubation temperature of cultures from 22 to 26°C did not induce any changes in morphology and weekly subcultures at these temperatures for 2 months showed that the morphological phenotype was a stable characteristic (data not shown).

### Identification of the EFSF6 isolate

Although the isolate was obtained from a patient with localized skin lesions and had been previously identified as *L. (V.) braziliensis *[13], the unusual morphology urged a more thorough identification assessment. *In vitro *cultured parasites were tested using a panel of 16 monoclonal antibodies described as tools for *Leishmania *identification and typing [14-16]. Positive reactions were detected with monoclonal antibodies L1, CO2, B12, B5 and B18, confirming the identification of a parasite belonging to the *Leishmania *genus and typing the isolate as *L. (V.) braziliensis*. Reactions with monoclonal N2, usually positive against the reference strain M2903, were negative with isolate EFSF6 (data not shown).

Molecular confirmation was also based on sequencing of the ribosomal internal transcribed spacers 1 and 2 and 5.8S rDNA. These molecular markers are widely used in the identification of trypanosomatids and can be employed to determine phylogenetic relationships and also assist in the identification of a particular isolate. A 985 nt ITS1/5.8S/ITS2 sequence was determined for the EFSF6 isolate. Sequence comparison over the conserved 5.8S sequence showed 100% identity with all other *Leishmania *sequences, as expected. Alignment of ITS1 and 2 segments indicated 97-98% identity with *L. (V.) braziliensis *and *L. (V.) guyanensis *sequences (data not shown). A maximum parsimony analysis grouped the EFSF6 isolate with *L. (V.) braziliensis *(Additional File 1).

### Detailed morphological characterization of *L. (V.) braziliensis *EFSF6 parasites

**Figure 3:**
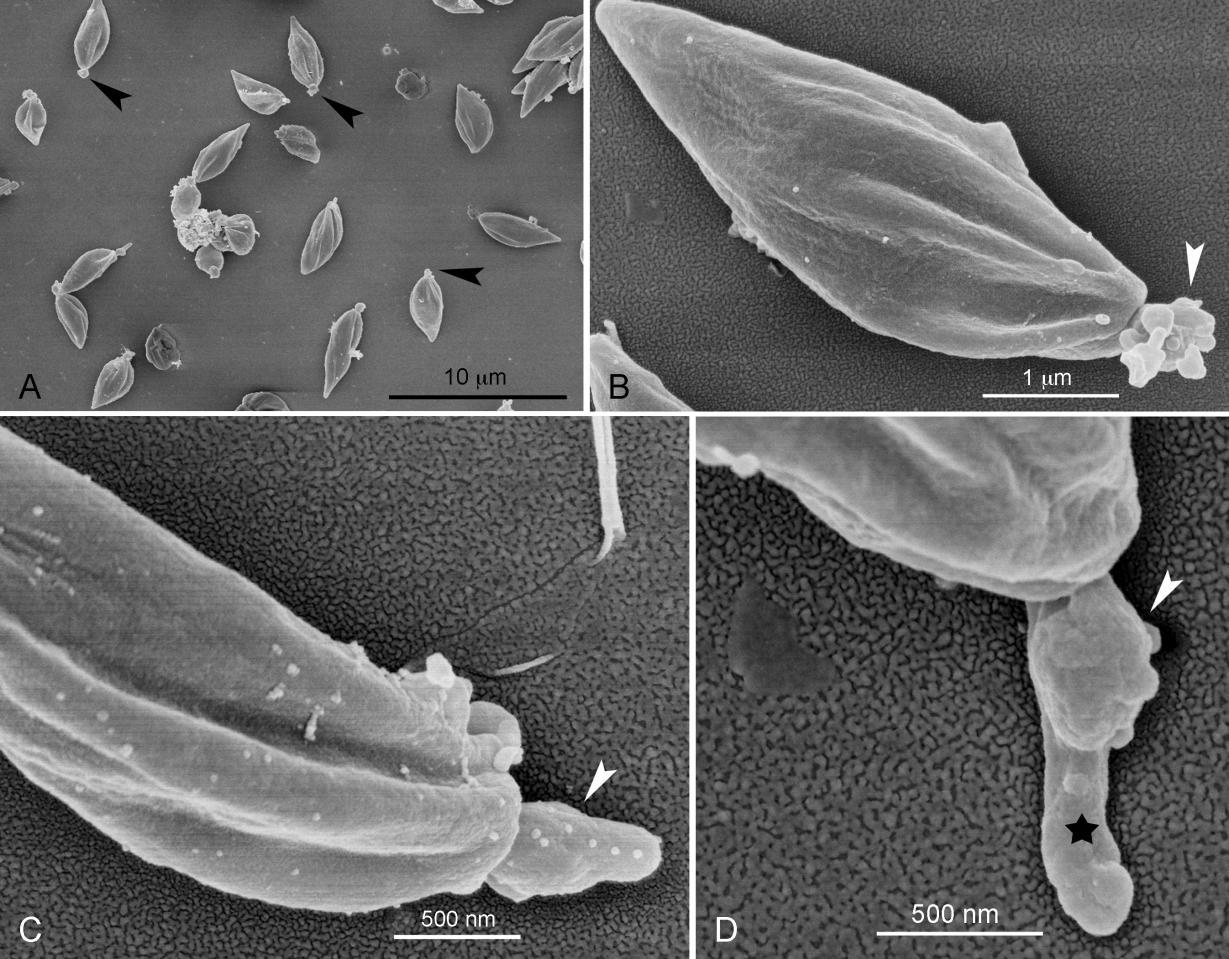
**Scanning electron microscopy of EFSF6 promastigotes**. (A) logarithmic phase promastigotes with atypical flagellum (arrow). (B) and (C) presence of longitudinal grooves and rounded micellar corpuscles (arrow). (D) anterior region (flagellum) of promastigotes.

Observation of EFSF6 promastigotes under scanning electron microscopy confirmed the fusiform or pyriform morphology of the cell bodies for most promastigotes (Figure 3A). The presence of longitudinal grooves, often twisted along the body of the parasite was noted. Over 85% of the logarithmic phase EFSF6 promastigotes showed the atypical flagellum surrounded or interrupted by a protuberance of disorganized material (Figure 3B-D, arrowheads). In addition, some parasites also presented one small flagellum externalised from the flagellar pocket that appeared associated with a disorganised structure (Figure 3D, star). Many parasites had vesicles adhered to the cell surface (Figure 3C).

**Figure 4:**
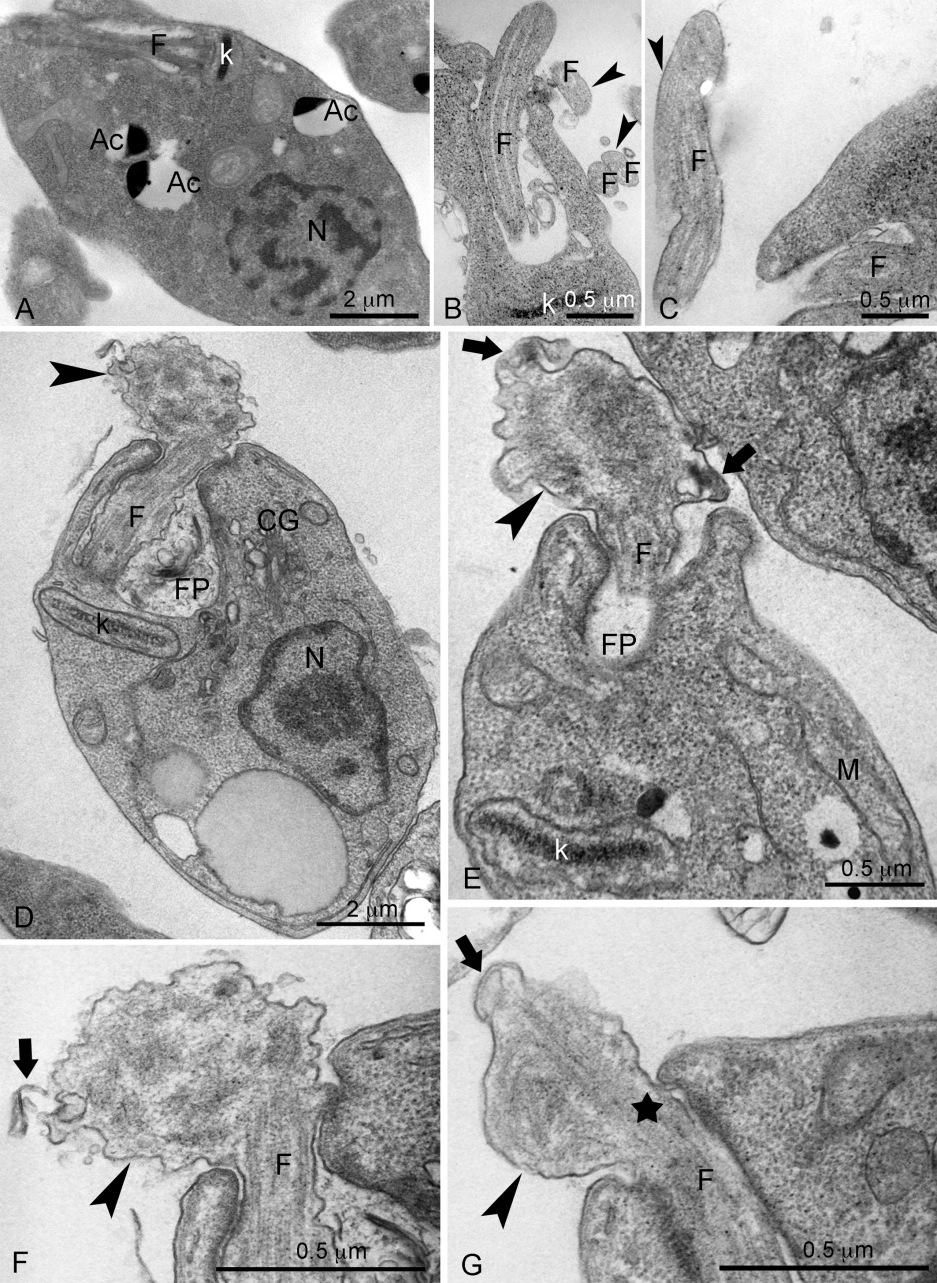
**Ultrathin section of promastigotes of the reference strain M2903 (A-C) and EFSF6 parasites (D-G)**. These images illustrate the ultrastructure of promastigotes from 3^rd ^day of culture analysed by transmission electron microscopy. (N) nucleus, (K) kinetoplast, (FP) flagellar pocket, (F) flagellum and (Ac) acidocalcissomes.

EFSF6 parasites along with the reference strain were also examined using transmission electron microscopy of fixed promastigotes. The M2903 promastigotes showed a typical flagellar structure formed by the axoneme and paraflagellar rod (Figure 4A-C, arrowhead). In a longitudinal section of M2903 promastigotes, it was possible to observe a normal kinetoplast present in the anterior region, the flagellar pocket, some acidocalcisomes and a nucleus. EFSF6 cells showed a typical single mitochondria containing the distinctive array of kinetoplast DNA (Figure 4D-E). The flagellar basal body with an erupting flagellum with normal structure was observed (Figure 4D-G). At the anterior extremity and where the flagellum is due to emerge from the flagellar pocket, a disorganized bulge (indicated by the arrowhead) was noted (Figure 4D-G). This disorganized swelling coincides with the position where the paraflagellar rod should be inserted. From that point towards the exterior of the cell, a disassembly of the axoneme was observed. In high magnification, it was also possible to detect a disorganization of the membrane that surrounds the abnormal flagellar structure (Figure 4F-G, arrow). In some cells the axoneme leaving the flagellar pocket showed a normal organization (Figure 4G, star), with features similar to those observed by scanning electron microscopy (Figure 3D, star). Acidocalcisomes (Ac) and lipid inclusions (stars) were also observed in the EFSF6 cells (Figure 4D-E).

### *In vitro *and *in vivo *infectivity of the EFSF6 isolate

**Figure 5:**
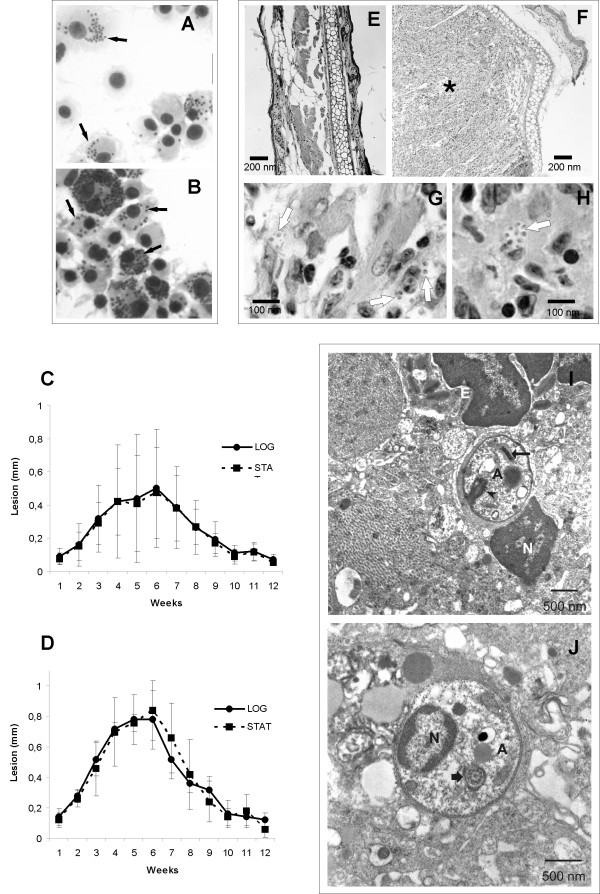
**Infectivity of EFSF6 promastigotes *in vitro *and *in vivo***. (A-B) Susceptibility of BMDM to infection with the EFSF6 isolate. Macrophages were infected with stationary-phase promastigotes *of L. (V.) braziliensis *reference strain (MHOM/BR/75/M2903) (A) or with the EFSF6 isolate (B) using a ratio of 15 parasites/macrophage for 3 hours at 33°C and 5% CO_2_. The infection was maintained for 6 days under these conditions (magnification 1000×). (C-J) Infectivity of EFSF6 promastigotes to mice. (C-D) Lesion progression after inoculation of 5 × 10^6 ^EFSF6 promastigotes in the hind footpad of BALB/c (C) or C57BL/6 (D) mice. Parasites were from the 2^nd ^(solid line) or 6^th ^day of culture (dashed line). Results shown are the mean and standard deviation of lesion size in groups of 6 mice. Lesion size is the difference between the thickness of infected and non-infected contralateral footpads. (E-H) Histopathological analysis of the uninfected (E) or EFSF6-infected (F-H) ear of a BALB/c mouse. Tissue was fixed 3 weeks after the inoculation of 1 × 10^5 ^EFSF6 promastigotes. Magnification: 40× (E-F) and 1000× (G-H). (I-J) Transmission electron microscopy showing ear fragments from mice infected with EFSF6. Arrows point to amastigotes surrounded by tight parasitophorous vacuoles; the thick arrow indicates a kinetoplast and the thin arrow shows the amastigote rudimentary flagellum. (A) amastigote, (N) nucleus, (E) eosinophil.

To test whether the parasites recovered were infective, we performed *in vitro *and *in vivo *infection experiments. The typical pattern of macrophage infection for *L. (V.) braziliensis *consists of small vacuoles with few amastigotes per vacuole (Figure 5A). EFSF6 promastigotes from *in vitro *culture were able to infect murine bone marrow derived macrophages, which became heavily parasitized (Figure 5B) with intracellular amastigotes distributed in tight vacuoles.

EFSF6 promastigotes were also used to infect BALB/c and C57Bl/6 mice. The injection of 5 × 10^6 ^parasites at the hind footpad of either mice lineage induced small lesions, apparent from week 3 and reaching their peak at week 5 or 6 (Figure 5C, D). By week 10 footpads showed no macroscopic signs of infection. Lesion progression was similar, irrespective of the day of culture of EFSF6 promastigotes inoculated (Figure 5C, D). Parasites isolated from mouse footpad lesions and cultured in Grace's medium maintained the same morphological characteristics of those parasites isolated directly from the patient fragment biopsy (data not shown).

BALB/c mice were also infected at the ear dermis. Swelling at the inoculation site was noted by week 3, peaked at week 6 and, by week 10, all animals were clinically cured (data not shown). Histopathological sections of the ear from infected mice showed a mononuclear inflammatory reaction and the presence of parasitized macrophages (Figure 5F-H). Biopsies of BALB/c ears infected with EFSF6 were also analysed by transmission electron microscopy. The examination of these tissue biopsies allowed the identification of typical amastigotes inside host cells, confirming the infectivity of the EFSF6 promastigotes. These parasites were contained inside tight vacuoles and exhibited the characteristic kDNA structure, being therefore indistinguishable from typical *Leishmania *amastigotes (Figure 5I-J).

### Infectivity of EFSF6 parasites to phlebotomine sand flies

**Figure 6:**
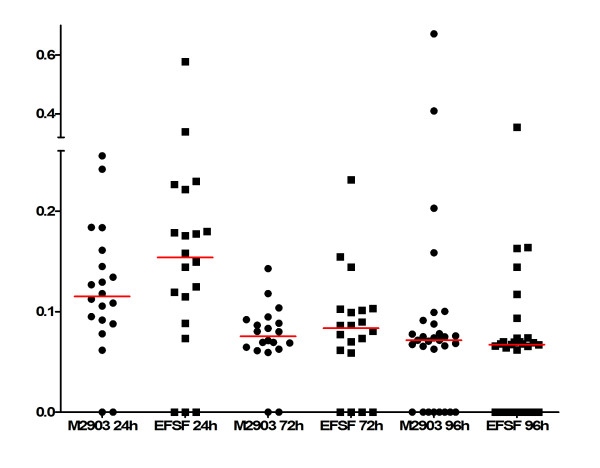
**Infection of *L. longipalpis *with *L. (V.) braziliensis *M2903 or EFSF6. Female sandflies (n = 140) were artificially infected with *L. (V.) braziliensis *M2903 (circles) or EFSF6 (squares)**. After 24, 48, 72 and 96 hours post infection *Leishmania *load was determined in at least 20 individuals per timepoint.

Lastly, we investigated whether phlebotomine sand flies could be successfully infected with EFSF6 parasites. To that purpose, female *L. longipalpis *were fed on hamster blood containing EFSF6 or M2903 *L. braziliensis *promastigotes. After various time intervals, DNA was prepared from individual flies and parasites were quantified by qPCR. Flies fed with the EFSF6 isolate were successfully infected, as shown by the presence of amplification 72 and 96 hours post-feeding (Figure 6). All flies analysed after 96 hours had already expelled the remains of the blood meal. There was no difference in the number of infected flies 24, 72 or 96 hours post-feeding EFSF6 or M2903 parasites. A small but not significant difference was observed at 48 h post-feeding. In particular, a comparable number of positive flies after 96 hours, whether infected with EFSF6 or M2903 parasites, indicated that both parasites survived proteolytic blood digestion and excretion of blood and peritrophic membrane remnants, being able to establish a successful infection in the insect vector.

## Discussion

In this paper we describe the morphological and biological characteristics of a parasite isolated from a cutaneous leishmaniasis patient. The morphology of this isolate was atypical ever since it was recovered *in vitro *from the tissue biopsy. Initially, the observation of the cultures raised doubts even as to whether it was indeed a *Leishmania *isolate. Identification methods using several different criteria, including monoclonal antibodies and genotyping by ribosomal RNA conserved and variable sequences led to a definite typing as *L. (V.) braziliensis*. To our knowledge, the morphological characteristics exhibited by EFSF6 parasites in culture medium that imitates the insect gut have not been previously described on any field isolate. Strictly speaking these parasites could not be called promastigotes since they do not exhibit a free anterior flagellum. In the course of this paper, however, we took the license to call them atypical promastigotes. They are able to grow in *in vitro *cultures and the phenotype is stable.

A morphological analysis by optical and electron microscopy suggested that these parasites exhibit a defective machinery for flagellum elongation. The basal body and initial portion of the axoneme are present but the axoneme does not elongate further after the exit from the flagellar pocket. The absence of the paraflagellar rod, characteristic of trypanosomatids is striking. At the emergence of this short axoneme, a deposit of material is found, suggesting that proteins synthesized to travel along the flagellum are trapped at this position.

The causes for this mutated phenotype are presently unknown. The paraflagellar rod contains 2 proteins, PFR1 and PFR2. The functional role of the paraflagellar rod in *L. (L.) mexicana *was evaluated using mutants lacking the PFR1 and PFR2 genes. Ablation of PFR1 and PFR2 proteins in *L. (L.) mexicana *promastigotes induced severe perturbations in flagellar wave propagation; however, the length of the axoneme was not changed in these null mutants [27,28]. In these paraflagellar rod null mutants, transmission electron microscopy revealed the presence of a disorganized material connected to the axoneme through thin filaments, indicating that these two proteins are essential for the assembly of the paraflagellar rod structure.

Several studies have revealed the presence of different proteins probably involved in the assembling of the flagellar structure in *Leishmania*. In *L. (L.) donovani*, the lack of a gene that encodes the 70 kDa subunit of the outer dynein arm docking complex exhibited shorter and rounded cell bodies and a pronounced shortening of the flagellar length with severe alterations in motility and a strongly reduced cellular growth. However, these parasites have a typical paraflagellar rod structure [29]. Ablation of genes encoding the dynein-2 heavy chain [30], ADF/cofilin [31], or MAP kinase proteins [32] in *L. (L.) mexicana *resulted in perturbation of cell motility, lack of the paraflagellar rod and disorganization of the axoneme. All these features are similar to those observed in the *L. (V.) braziliensis *EFSF6 promastigotes, which underlines the complexity of the machinery responsible for assembling the flagellar and paraflagellar structures. Other proteins, such as a thymidine kinase and mitogen-activated protein kinase have been implicated in normal elongation of the flagellum and in this case the lack of the protein results in a lengthened flagellum [33,34].

The flagellum of promastigotes has been implicated in macrophage invasion by promastigotes in *L. (L.) donovani *[8]. In spite of the striking morphological differences, promastigotes of the EFSF6 isolate were infective *in vitro *to BMDM as well as to peritoneal macrophages (data not shown). Furthermore, inoculation of EFSF6 promastigotes into C57Bl/6 or BALB/c mice gave rise to disease patterns indistinguishable from other *L. (V.) braziliensis *strains, which induce a self-healing disease in these experimental models. However, the question of whether this isolate was transmitted to the patient as such is elusive.

On the other hand, a relationship between an intact flagellum and survival in the insect vector has been observed [6] (and references therein). An intact flagellum is obviously important for motility, which should serve a function in the necessary migrations the parasite has to perform while inside the fly. Furthermore, the attachment via flagellum to the epithelial surface seems to be important. In fact, an aflagellated *L. (L.) amazonensis *mutant has been shown to be unable to infect *L. longipalpis *[7].

*L. longipalpis *is not the natural vector of *L. (V.) braziliensis*, but it is a permissive vector, supporting the growth of several *Leishmania *species, including *L. (V.) braziliensis*. It has been suggested that in specific Old World vectors *Leishmania *surface lipophosphoglycan is determinant to successful attachment to the gut, while in permissive vectors other factors, including the interaction with the flagellum gain importance [35]. The natural infection of sand flies occurs by the ingestion of amastigote-containing blood but they can be infected with promastigotes [36]. The infection of *L. longipalpis *with the EFSF6 isolate showed that these atypical promastigotes are perfectly able to survive in the fly for the first 96 hours, or, in other words, after the expulsion of the digested blood and degraded peritrophic membrane. It will also be of utmost interest to find out whether these parasites can be transmitted by the bite of the phlebotomine sand fly, which could give us some indication about the likelihood of finding this type of mutated parasites in the field.

The EFSF6 isolate opens several possibilities to a new understanding of the roles of the flagellum in the life cycle, transmission and pathogenesis. We still do not know what is missing in this mutant and several approaches are being considered to investigate that. Meanwhile, we believe this isolate demonstrates that the variability and infectious properties of *Leishmania *in the field have not been completely understood and uncovered.

## Conclusions

We have characterized a clinical isolate of *L. (V.) braziliensis *that presents as a natural dysflagellar mutant, where the axoneme is present but unable to elongate to constitute an external flagellum. In spite of that, these parasites are able to infect macrophages *in vitro*, cause disease in mice and infect phlebotomine sand flies. This isolate may be able to provide valuable information about the infectious processes used by the parasite both in the vertebrate and invertebrate hosts.

## Competing interests

The authors declare that they have no competing interests.

## Authors' contributions

ATB, MLD, FRD, SRBU conceived the study; RCZ, JKUYY, DCM, LIAP, IASJ, LGNL, MAPO, EI worked in the isolation of the parasite, *in vitro *and *in vivo *characterization; RCZ, DCM, ASM, JCFR, ATB, worked on the morphological characterization of the parasite; ANP, YMTC performed the sand fly work; ATB, FRD, YTC and SRBU analyzed the data and wrote the manuscript. All authors read and approved the final version of the manuscript.

## Supplementary Material

Additional file 1 **Molecular identification and phylogenetic relationships of the EFSF6 isolate**. Dendrogram based on ITS1/5.8S/ITS2 sequences from the following organisms: *L. (L.) braziliensis *EFSF6 [GenBank JQ061322], *L. (L.) chagasi *[GenBank AJ000305.1], *L. (L.) donovani *[GenBank AJ000293.1, AM901450.1], *L. (L.) major *[GenBank AJ000310.1, DQ300195], *L. (L.) amazonensis *[GenBank AJ000314], *L. (L.) mexicana *[GenBank AF466383, AF466380.1], *L. (V.) guyanensis *[GenBank FJ753387, AJ000299.1, AJ000300.1], *L. (V.) braziliensis *[GenBank AJ300483, AJ300484, AJ300483] e *Leptomonas mirabilis *[GenBank AY180153.1]. The numbers at the branches refer to parsimony percentage bootstrap values derived from 100 replicates. Click here for file

Additional file 2 **Phase contrast imaging of *L. (V.) braziliensis *M2903 promastigotes in M199 medium**. Representative 30 seconds video, taken at 72 h of culture started with an inoculum of 1 × 10^6 ^parasites/ml. This movie shows the general movement of typical *L. (V.) braziliensis *promastigotes. Note the fast moving parasites that travel across the field and the typical rosettes. Click here for file

Additional file 3 **Phase contrast imaging of *L. (V.) braziliensis *EFSF6 promastigotes in M199 medium**. Representative 30 seconds video, taken at 72 h of culture started with an inoculum of 1 × 10^6 ^parasites/ml. This movie shows that these parasites tend to gather in groups. No fast movement is observed. A vibration is noted on the clusters of cells. Click here for file
