# Supplementary figures and images for: A dysflagellar mutant of Leishmania (Viannia) braziliensis isolated from a cutaneous leishmaniasis patient
Source: Parasit Vectors. 2012 Jan 11;5:11. doi: 10.1186/1756-3305-5-11 (PMC3271977; doi:10.1186/1756-3305-5-11)

## Slide 1
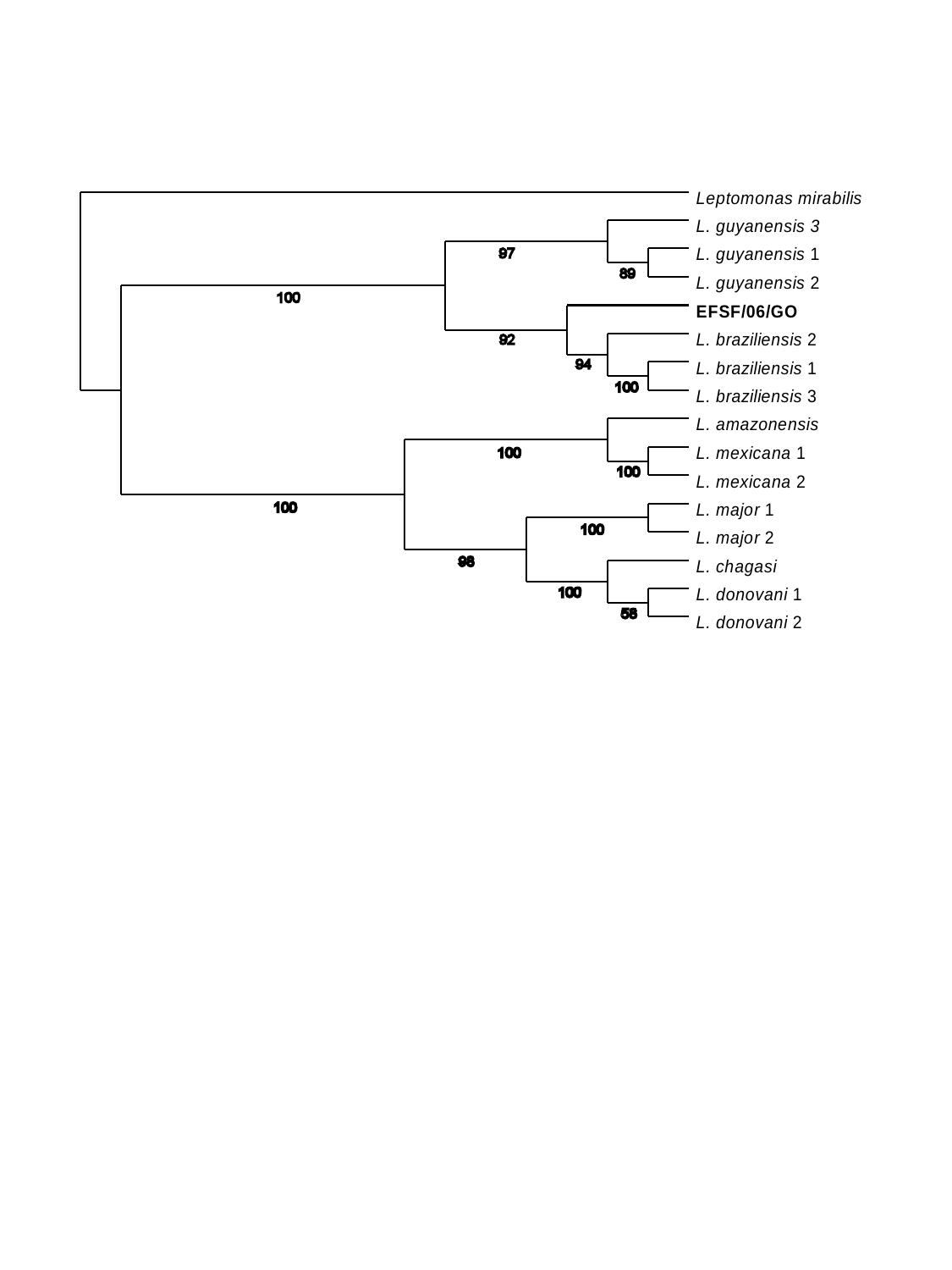

Supplement: Additional file 1 — Molecular identification and phylogenetic relationships of the EFSF6 isolate. Dendrogram based on ITS1/5.8S/ITS2 sequences from the following organisms: L. (L.) braziliensis EFSF6 [GenBank JQ061322], L. (L.) chagasi [GenBank AJ000305.1], L. (L.) donovani [GenBank AJ000293.1, AM901450.1], L. (L.) major [GenBank AJ000310.1, DQ300195], L. (L.) amazonensis [GenBank AJ000314], L. (L.) mexicana [GenBank AF466383, AF466380.1], L. (V.) guyanensis [GenBank FJ753387, AJ000299.1, AJ000300.1], L. (V.) braziliensis [GenBank AJ300483, AJ300484, AJ300483] e Leptomonas mirabilis [GenBank AY180153.1]. The numbers at the branches refer to parsimony percentage bootstrap values derived from 100 replicates. [file 1756-3305-5-11-S1.PPT]
